# The small G protein Arl8 contributes to lysosomal function and long-range axonal transport in *Drosophila*

**DOI:** 10.1242/bio.035964

**Published:** 2018-08-20

**Authors:** Cláudia Rosa-Ferreira, Sean T. Sweeney, Sean Munro

**Affiliations:** 1 MRC Laboratory of Molecular Biology, Francis Crick Avenue, Cambridge, CB2 0QH, UK; 2 Department of Biology, University of York, York, YO10 5DD, UK

**Keywords:** Arl8, *Drosophila*, RILP, Axonal transport, Dynein, Lysosome

## Abstract

The small GTPase Arl8 has emerged as a major regulatory GTPase on lysosomes. Studies in mammalian cells have shown that it regulates both fusion with late endosomes and also lysosomal motility. In its active GTP-bound state, it recruits to lysosomes the HOPS (homotypic fusion and protein sorting) endosomal tethering complex and also proteins that link lysosomes to microtubule motors such as the kinesin adaptor PLEKHM2. To gain further insights into Arl8 biology, we examined the single *Drosophila* ortholog. *Drosophila* Arl8 is essential for viability, and mitotic clones of mutant cells are able to continue to divide but show perturbation of the late endocytic pathway. Progeny-lacking Arl8 die as late larvae with movement-paralysis characteristic of defects in neuronal function. This phenotype was rescued by expression of Arl8 in motor neurons. Examination of these neurons in the mutant larvae revealed smaller synapses and axons with elevated levels of carriers containing synaptic components. Affinity chromatography revealed binding of *Drosophila* Arl8 to the HOPS complex, and to the *Drosophila* ortholog of RILP, a protein that, in mammals, recruits dynein to late endosomes, with dynein being known to be required for neuronal transport. Thus *Drosophila* Arl8 controls late endocytic function and transport via at least two distinct effectors.

This article has an associated First Person interview with the first author of the paper.

## Abstract (precis)

**Summary:** *Drosophila* Arl8 is essential for viability and is required for normal functioning of the late endocytic pathway and for long-range transport in axons.

## INTRODUCTION

Small GTPases of the Rab and Arf families are major regulators of the function of all of the compartments of the secretory and endocytic pathways (Gillingham and Munro, 2007; Hutagalung and Novick, 2011). In the case of lysosomes, the primary lysosome-specific GTPase is Arl8, a member of the Arf family that is conserved in most eukaryotic phyla with the exception of a few lineages such as budding yeasts (Hofmann and Munro, 2006; Khatter et al., 2015b). In vertebrates there are two closely related paralogs Arl8a and Arl8b, of which Arl8b is generally more abundant. Activation of Arl8a and Arl8b requires a large protein complex called BORC [biogenesis of lysosome-related organelles complex (BLOC)-one-related complex], and it has been proposed that this acts as an Arl8 exchange factor (Niwa et al., 2017; Pu et al., 2015). Several effectors have been identified that are recruited to lysosomal membranes by the active GTP-bound form of Arl8a and Arl8b. A well-established effector in mammalian cells is the HOPS (homotypic fusion and protein sorting) complex, a multisubunit complex that contains a member of the SM family of SNARE-activating proteins and is known to be required for various fusion steps between compartments in the late endocytic pathway (Balderhaar and Ungermann, 2013; Garg et al., 2011; Khatter et al., 2015a). A second effector in mammals is SKIP/PLEKHM2, a linker protein that then recruits kinesin-1 to lysosomal membranes (Rosa-Ferreira and Munro, 2011). In addition, the *Caenorhabditis elegans* ortholog Arl-8 binds directly to kinesin-3 and, in mammals, Arl8a and Arl8b act upstream of kinesin-3 (Guardia et al., 2016; Wu et al., 2013).

Thus, Arl8 activity regulates both lysosome fusion and lysosome motility and, consistent with this, Arl8 is required for a range of biological processes that require one or both of these activities. These include lysosome positioning and morphology, delivery of proteins to lysosomes, fusion of lysosomes to phagosomes, long-range transport in axons, and relocation of lysosome-related organelles in cytotoxic T cells (Dykes et al., 2016; Garg et al., 2011; Kaniuk et al., 2011; Klassen et al., 2010; Mrakovic et al., 2012; Nakae et al., 2010). This dual function in lysosome function and lysosome location is also consistent with recent reports indicating that these two properties of lysosomes are tightly linked (Bonifacino and Neefjes, 2017; Johnson et al., 2016).

Whilst Arl8 has emerged as a master regulator of lysosomal function in cells, it has also become clear that lysosomes themselves have a broader biological role than simply turning over cellular components. Lysosomes have been shown to be the site at which metabolic status is linked to cell growth, with the mTORC1 pathway being regulated on the lysosomal membrane (Efeyan et al., 2012; Perera and Zoncu, 2016). Indeed, the Ragulator complex that activates mTORC1 on lysosomal membranes also regulates BORC and thus controls Arl8 activity and hence lysosomal positioning (Filipek et al., 2017; Pu et al., 2017).

Given the importance of lysosomes for diverse cellular processes, and the key role that Arl8 plays in regulating lysosomal function, it seems likely that there is more to be learned about the role of Arl8. In particular there have been relatively few genetic studies of Arl8 function. Mouse embryos lacking Arl8b develop abnormally and the mice die just before or shortly after birth, with there being major defects in lysosomal processing of maternal proteins by the visceral yolk sac endoderm that takes up nutrients from the uterine fluid (Oka et al., 2017). In *C. elegans*, the single Arl8 ortholog, ARL-8, is essential for viability. Homozygous mutant worms born to heterozygous mothers develop to adulthood, but cannot produce viable embryos and show defects in endocytosis, phagolysosome formation and long-range transport in axons (Klassen et al., 2010; Nakae et al., 2010; Sasaki et al., 2013). We have extended these genetic studies by examining the role of the single Arl8 ortholog in *Drosophila melanogaster*. The gene that encodes the protein is CG7891 and it was initially referred to as *Gie* (for ‘GTPase indispensable for equal segregation of chromosomes’) based on an early report that mammalian and *Drosophila* Arl8 may be involved in chromosome segregation with the mammalian protein reported to be located on the mitotic spindle of PC12 cells (Okai et al., 2004). However, this location for Arl8 or such a role in chromosome segregation has not been observed in any subsequent study, including those from the authors of the original report (Nakae et al., 2010). Thus, for the sake of clarity and consistency, we will refer to the *Drosophila* gene as *Arl8*. The *Drosophila* Arl8 protein has been shown to localise to lysosomes in *Drosophila* S2 cultured cells, and when expressed in mammalian cells it is also recruited to lysosomes, suggesting that it is functionally similar to mammalian Arl8 proteins (Hofmann and Munro, 2006). Here we examine the phenotypes of a *Drosophila* mutant lacking detectable Arl8 protein, and go on to use affinity chromatography to identify effectors for *Drosophila* Arl8. Our findings indicate that Arl8 has a similar role to that described in mammals and *C. elegans*, and in addition provide evidence for a direct interaction with RILP, a linker to the dynein microtubule motor.

## RESULTS

### Arl8 is essential for viability

**Fig. 1.**
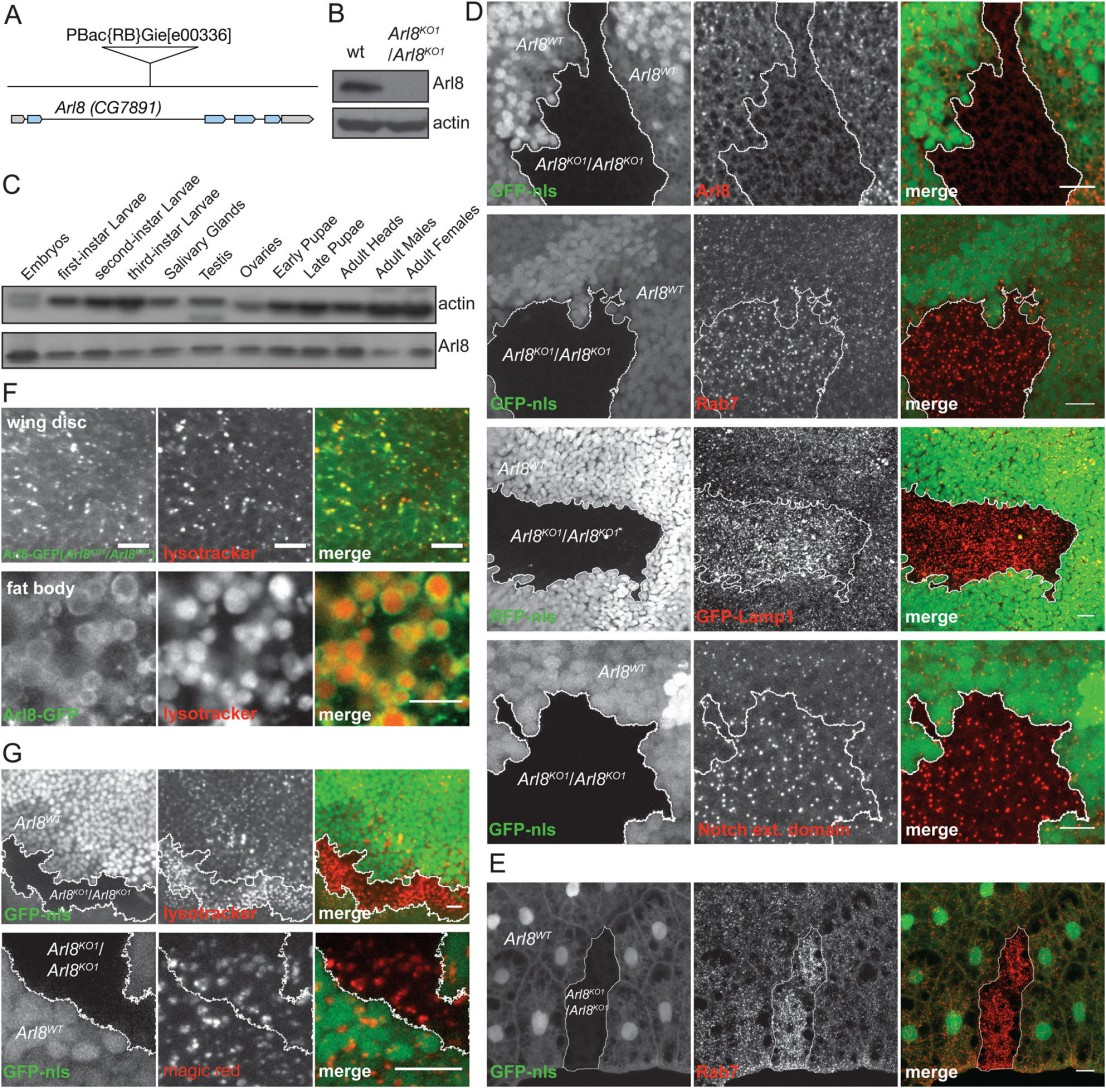
**Deletion of the *Drosophila* ortholog of Arl8 affects the late endocytic pathway.** (A) Schematic illustration of the *Arl8* gene (CG7891) showing the location of the PiggyBac element PBac{RB}Gie^e00336^. The grey areas indicate untranslated regions and blue areas indicate the coding region of *Arl8*. (B) Immunoblot of lysates from L3 stage larvae from wild-type (wt, w^1118^) and w^1118^; PBac{RB}Gie^e00336^/PBac{RB}Gie^e00336^, Tb^1^ probed for Arl8 and for β-actin as a control. (C) Immunoblot of lysates of a range of fly tissues and of various development stages. The blots were probed for Arl8 and for β-actin. (D,E) Confocal micrographs of wing imaginal discs (D, scale bars: 10 μm) or of fat body (E, scale bar: 20 μm) from L3 larvae where mosaic clones were generated by mitotic recombination. The *Arl8^KO1^/Arl8^KO1^* mutant clones are marked by the absence of nuclear GFP or RFP, which also indicates that each clone contains many cells of the tissue. Arl8, Rab7 and Notch extracellular domain were detected with specific antibodies, and the fly lines used to generate were: P{hsFLP}22, y^−^w^−^; +/+; P{neoFRT}82B, P{GFPnls}/TM6B, Hu Tb and P{hsFLP}22, y^−^w^−^; +/+; P{neoFRT}82B, PBac{RB}Gie^e00336^/TM6B, e Hu Tb. GFP-Lamp1 was expressed under the control of the Act5C promoter using fly lines: P{hsFLP}22, y^−^w^−^; P{W^+^ UAS-GFP Lamp1}/Cyo; P{neoFRT}82B, PBac{RB}Gie^e00336^/ TM6B, e Hu Tb; and P{hsFLP}22, y^−^w^−^; act5C-GAL4/CyO; P{neoFRT}82B, Ubi P{mRFPnls}/ TM6B. In all cases where mitotic clones are shown at least 10 different clones were imaged and representative examples are shown. (F) Confocal micrographs of wing imaginal discs and of fat body from L3 larvae. On the first row, Arl8-GFP was expressed in the *Arl8^KO1^/Arl8^KO1^* mutant, stained live with LysoTracker. The lines used were w^−^; P{W^+^ UAST-Arl8 GFP}/Cyo; PBac{RB}Gie^e00336^/TM6B and w^−^; act5C-GAL4/CyO; PBac{RB}Gie^e00336^/TM6B. The second row is as the first, except that Arl8 is expressed in flies where endogenous Arl8 is present. (G) Clones in wing imaginal discs generated as in (D) and stained live with LysoTracker or Magic Red as indicated. Scale bars: 10 µm (D-G).

To examine the role of Arl8 in *Drosophila*, we initially investigated the consequence of removing Arl8 from flies. We examined a *Drosophila* stock that has a PiggyBac transposon PBac{RB}Gie^e00336^ inserted in the second intron of *Arl8* (Fig. 1A). This insertion is a recessive lethal, with no homozygous mutants developing beyond late L3 larval/early pupal stages. The lethality is due to the perturbation of the *Arl8* locus as it was also seen in heterozygotes of the PiggyBac insertion allele over a genomic deficiency (Df(3R)D7, 84D3-5;84F1-2) that encompasses the *Arl8* gene. In addition, the lethality can be rescued by expression of Arl8 or Arl8-GFP as described below. Arl8 protein was undetectable in lysates prepared from homozygous PBac{RB}Gie^e00336^ L3 larvae (Fig. 1B), and so for simplicity we shall refer to this apparent null allele as *Arl8^KO1^.*

### Loss of Arl8 affects late endocytic compartments

Arl8 is ubiquitously expressed in *Drosophila*, judged by there being comparable levels of Arl8 in various tissues and at various developmental stages (Fig. 1C). When FRT *Arl8^KO1^*was used in combination with the germline-dependent dominant female sterile *Ovo^D^* mutation (FRT ovo^D^), to remove Arl8 from the germline, the *Arl8^KO1^* mutant progeny did not reach larval stages and instead died at a late embryonic stage. This indicates that the maternal contribution of Arl8 accounts for the survival of the *Arl8^KO1^* homozygous progeny until larval/early pupal stage and that Arl8 is required for *Drosophila* embryonic development. To examine the effect of removing Arl8 from patches of cells later in development, we used the FRT system to generate mitotic clones of cells lacking Arl8 in a wild-type background. Thus we generated *Arl8^KO1^/Arl8^KO1^* mitotic clones in wing imaginal discs and fat body, with the absence of nuclear GFP in the mutant clones accompanied by the absence of endogenous Arl8 (Fig. 1D).

When we analysed the late endocytic compartment markers Rab7 and Lamp1 in *Arl8^KO1^/Arl8^KO1^* clones, we found that these compartments appeared larger in the clones compared to the surrounding heterozygous cells in both wing discs and fat body (Fig. 1D,E). In addition, a marker for endocytic protein clearance, the extracellular domain of Notch, which is continuously internalised and either recycled back to the plasma membrane or targeted for degradation in lysosomes (Fortini and Bilder, 2009), was found to accumulate in the cells in *Arl8^KO1^/Arl8^KO1^* clones in wing discs (Fig. 1D). These phenotypes are reminiscent of those reported for clones lacking the HOPS subunit *car* (Vps33a) which accumulate Rab7-positive compartments with internalised ligands and receptors such as Notch (Akbar et al., 2009). This would thus be consistent with Arl8 interacting with the HOPS complex to facilitate fusion between late endosomes and lysosomes, with the result that in the mutant clones Notch flux to the lysosome, and hence its degradation is impaired. This reduced degradation does not appear to reflect loss of acidification or lysosomal hydrolase activity – the dye LysoTracker accumulates in acidified compartments and in wild-type tissue co-localises well with Arl8 as expected (Fig. 1F). In mutant clones lacking Arl8, LysoTracker labelling is actually elevated compared to wild-type tissue, consistent with the findings described above for the late endocytic compartment markers Rab7 and Lamp1 (Fig. 1G). In addition, we could still observe staining with the reporter Magic Red that detects activity of the lysosomal hydrolase cathepsin B (Fig. 1G). This indicates that active hydrolases still accumulate in acidified compartments in the mutant clones. This would help maintain some digestive function, and could explain why the mutant clones are able to grow from the originating division to large patches of cells lacking Arl8, a process which could only occur if the cells are able to continue to grow and divide in the absence of Arl8.

### Neuronal function is impaired in the absence of Arl8

Mutations in the *C. elegans* gene encoding the ortholog of Arl8 result in defects in long-range transport in axons (Klassen et al., 2010; Niwa et al., 2016). This led us to look for evidence that *Drosophila* Arl8 also plays a role in microtubule-based transport *in vivo*. Indeed, we had noticed that in *Arl8^KO1^/Arl8^KO1^* progeny the movement of L3 larvae was limited (Movie 1), with posterior paralysis resulting in a characteristic tail flipping phenotype that resembles that reported for kinesin-1, dynein and dynactin mutants (Hurd and Saxton, 1996; Martin et al., 1999; Saxton et al., 1991). We thus tested if movement could be rescued when expression of Arl8 or of Arl8-GFP was driven using the Gal4 UAS system. We found that the Gal4 drivers Act5C (ubiquitous), nSyb (pan neural) and C164 (motor neurons), rescued full movement, but the Mef2 (muscle) driver still had reduced movement (Movie 2). Larvae with rescued movement reached adulthood but could not fly. These results suggest that it is Arl8 function in motor neurons that is essential for homozygous *Arl8^KO1^/Arl8^KO1^* progeny to reach adulthood.

### Arl8 is required for normal axonal transport

**Fig. 2.**
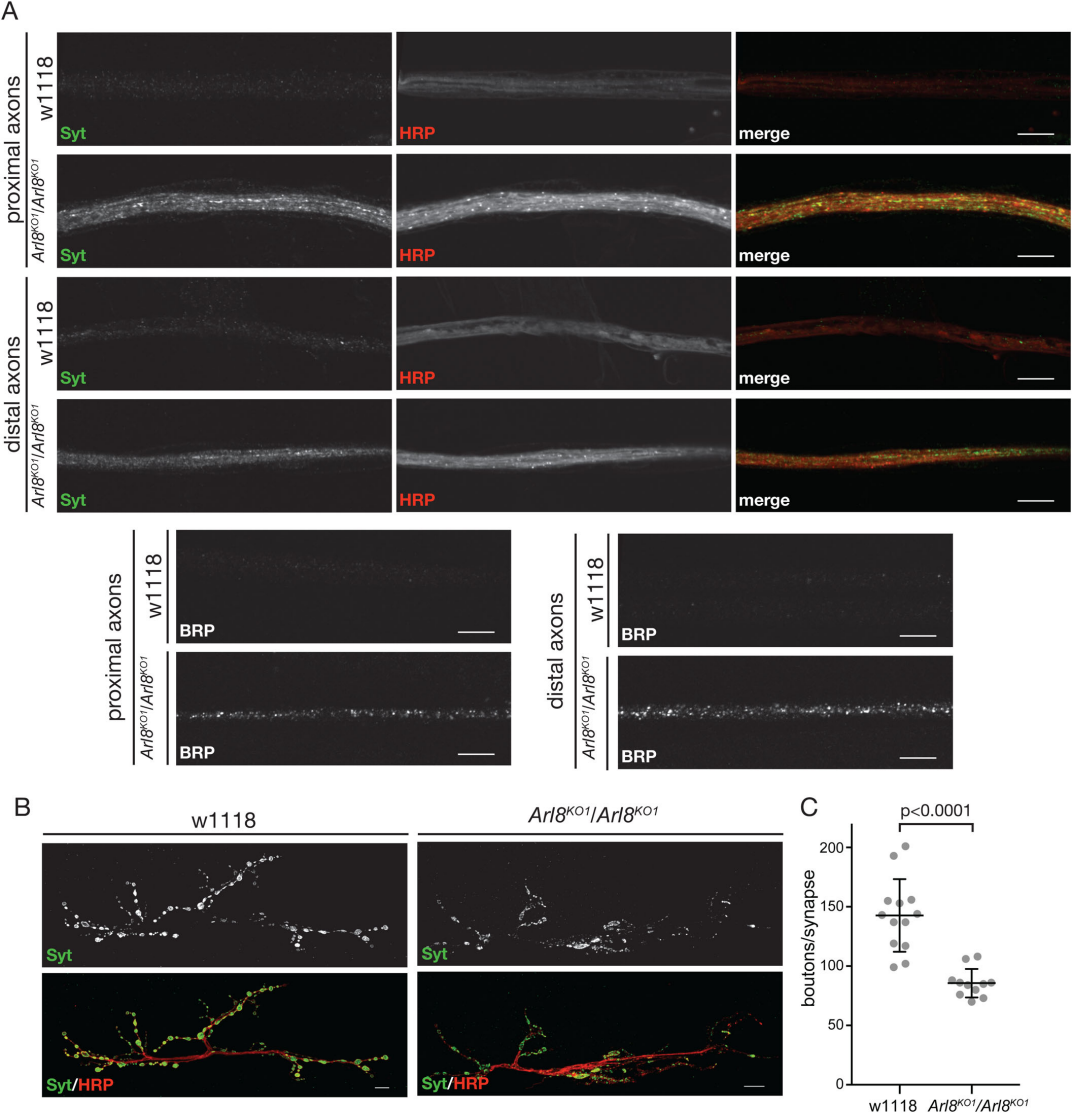
**Deletion of Arl8 affects long-range transport in axons.** (A) Confocal micrographs of proximal (segments A2/A3) and distal axons (segments A5/A6) from w^1118^ and *Arl8^KO1^/Arl8^KO1^* (w-; +/+; PBac{RB}Gie^e00336^/ PBac{RB}Gie^e00336^) larvae, stained with antibodies to HRP and to Syt1 (Synaptotagmin 1) or BRP (Bruchpilot) as indicated. Images are representative of those obtained from four different animals for each genotype, and in each case imaging was performed using the same settings for w1118 and mutant. Scale bars: 10 μm. (B) Confocal micrographs of active zones at the neuromuscular junction of the muscle 6/7, abdominal segment A2, from w^1118^ and *Arl8^KO1^/Arl8^KO1^* female larvae stained with anti-HRP and anti-Syt1 antibodies. Scale bars: 10 μm. (C) Quantification of the number of synaptic boutons (Syt1) from wild-type w^1118^ (*n*=13) and *Arl8^KO1^/Arl8^KO1^* (*n*=10) female larvae (muscle 6/7, abdominal segment A2). Mean and standard deviations are shown, with the reduction in the mutant being statistically significant (*P*<0.0001, two-tailed nonparametric Mann–Whitney test).

Axons are believed to lack a functional secretory pathway and so synaptic membrane proteins and their associated factors are transported from the cell body along the axon in carrier vesicles. To determine if loss of Arl8 results in defects in this long-range axonal transport we stained axons for the synaptic vesicle marker Synaptotagmin 1 (Syt1) and for the presynaptic active zone protein Bruchpilot (BRP). In *Arl8^KO1^/Arl8^KO1^* we observed elevated levels of these proteins in axons along with increased labelling with anti-HRP which detects neuronal membrane proteins (Fig. 2A). A similar accumulation of synaptic markers along axons has been reported for loss-of-function mutants in dynein heavy chain and kinesin-1, and for dominant negative mutations in dynactin subunits (Butzlaff et al., 2015; Füger et al., 2012; Gindhart et al., 1998; Hurd and Saxton, 1996; Martin et al., 1999). Another indication of defects in axonal transport of synaptic components was that synapses in axons of *Arl8^KO1^/Arl8^KO1^* larvae appeared smaller than in the wild-type. Quantification of the number of boutons per synapse, as marked by Synaptotagmin 1, revealed a 40% reduction in the number of synaptic boutons per synapse in the *Arl8* mutant (Fig. 2B,C). Taken together, these results indicate that Arl8 is required for normal long-range axonal transport.

### Arl8 moves along the axon to the synapse

**Fig. 3.**
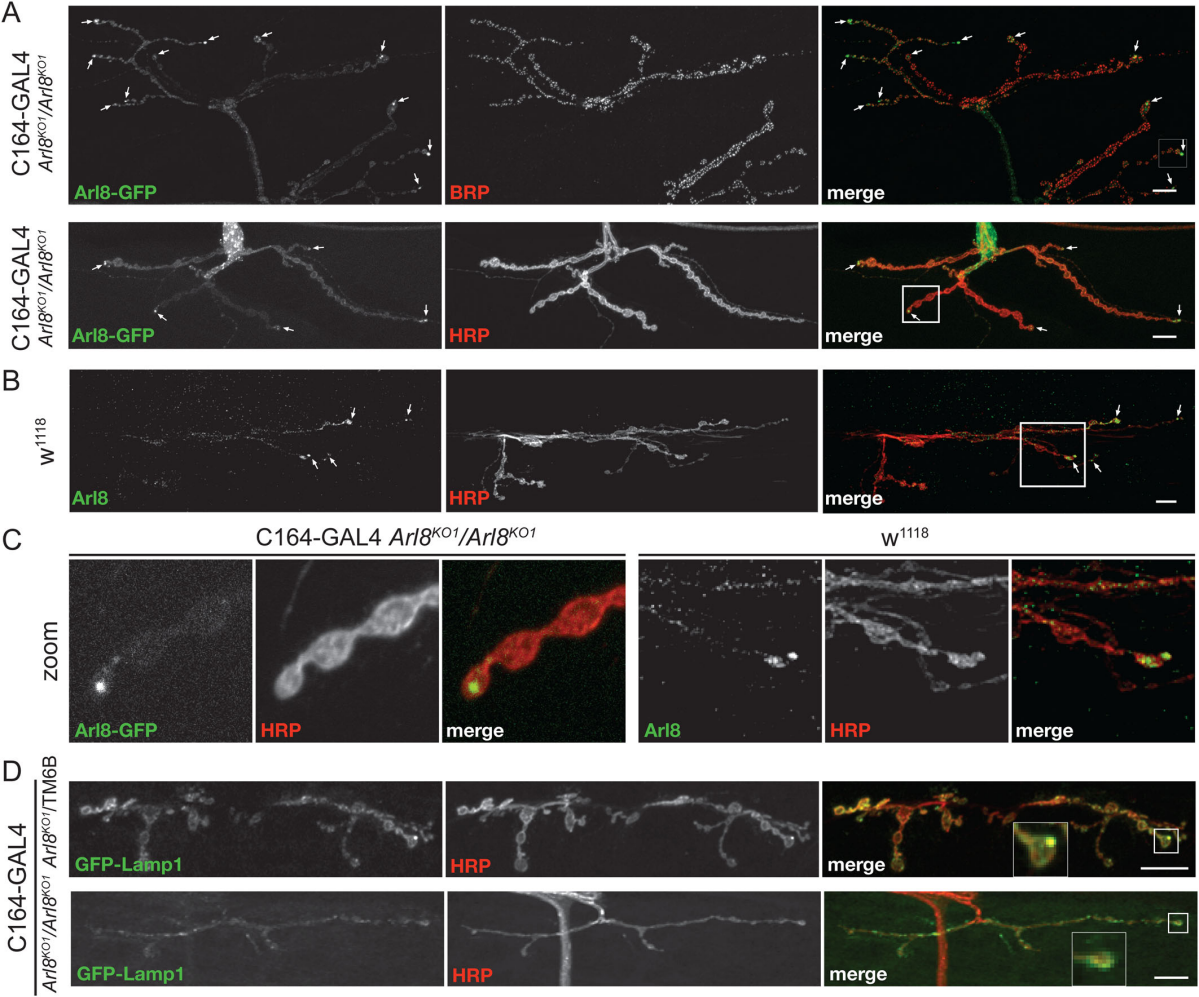
**Arl8 is present in axons and synapses.** (A) Confocal micrographs of synapses from flies expressing Arl8-GFP under control of the motor neuron-specific driver C164-Gal4, in an *Arl8^KO1^/Arl8^KO1^* mutant background, and stained with Bruchpilot specific antibodies (BRP) or with HRP antibodies to outline axons. Arrows indicate enrichment of Arl8-GFP in distal boutons. A square in the HRP merge image indicates a region of the synapse that is shown enlarged in (C). The flies used were w-; C164-GAL4/ CyO; PBac{RB}Gie^e00336^/ TM6B and w-; pUAST-Arl8GFP/CyO; PBac{RB}Gie^e00336^/ TM6B. In each case images were obtained from six animals and representative examples are show. Scale bars: 10 μm. (B) Arl8 localisation in synapses is shown by co-staining wild-type (w^1118^) synapses for endogenous Arl8 and with anti-HRP. Arrows indicate Arl8 in distal boutons. A square in the merge image indicates a region of the synapse shown enlarged in (C). Images are representative of those obtained from six animals. Scale bars: 10 μm. (C) Enlarged region of the synapses, indicated by squares in the merge images in A and B, showing the enrichment of endogenous Arl8 and of Arl8-GFP on the most terminal boutons. (D) Confocal micrographs of synapses expressing GFP-Lamp1 driven by C164 and stained for HRP, from control and *Arl8^KO^* mutant flies, heterozygous and homozygous for the *Arl8^KO1^* mutation, respectively. The lines used were w-; C164-GAL4/ CyO; PBac{RB}Gie^e00336^/ TM6B and w-; P{W^+^ UAS-GFP Lamp1}/CyO; PBac{RB}Gie^e00336^/ TM6B. Images were obtained from five (*Arl8^KO^*/TM6B) or three (*Arl8^KO^*/*Arl8^KO^*) animals, and representative images shown. Scale bars: 10 μm.

If Arl8 is contributing to long-range transport in *Drosophila* axons then the protein should be present in axons. To address this question, we stained nerve terminals with anti-Arl8 or expressed Arl8-GFP in motor neurons with the C164-Gal4 driver in an *Arl8^KO1^/Arl8^KO1^* background. In both cases, Arl8 staining was relatively faint but it could be detected in synapses, where it is apparently enriched in the most distal boutons, the place where microtubule plus-ends are located (Fig. 3A-C). We observed a similar distribution for GFP-Lamp1 (Fig. 3D), consistent with previous evidence that lysosomes are present in *Drosophila* synapses (Dermaut et al., 2005; Shen and Ganetzky, 2009; Sweeney and Davis, 2002). This distal accumulation of GFP-Lamp1 is absent in the *Arl8* mutant, again consistent with Arl8 being required for long-range transport to this location (Fig. 3D).

### Identification of interaction partners for *Drosophila* Arl8 using affinity chromatography

**Fig. 4.**
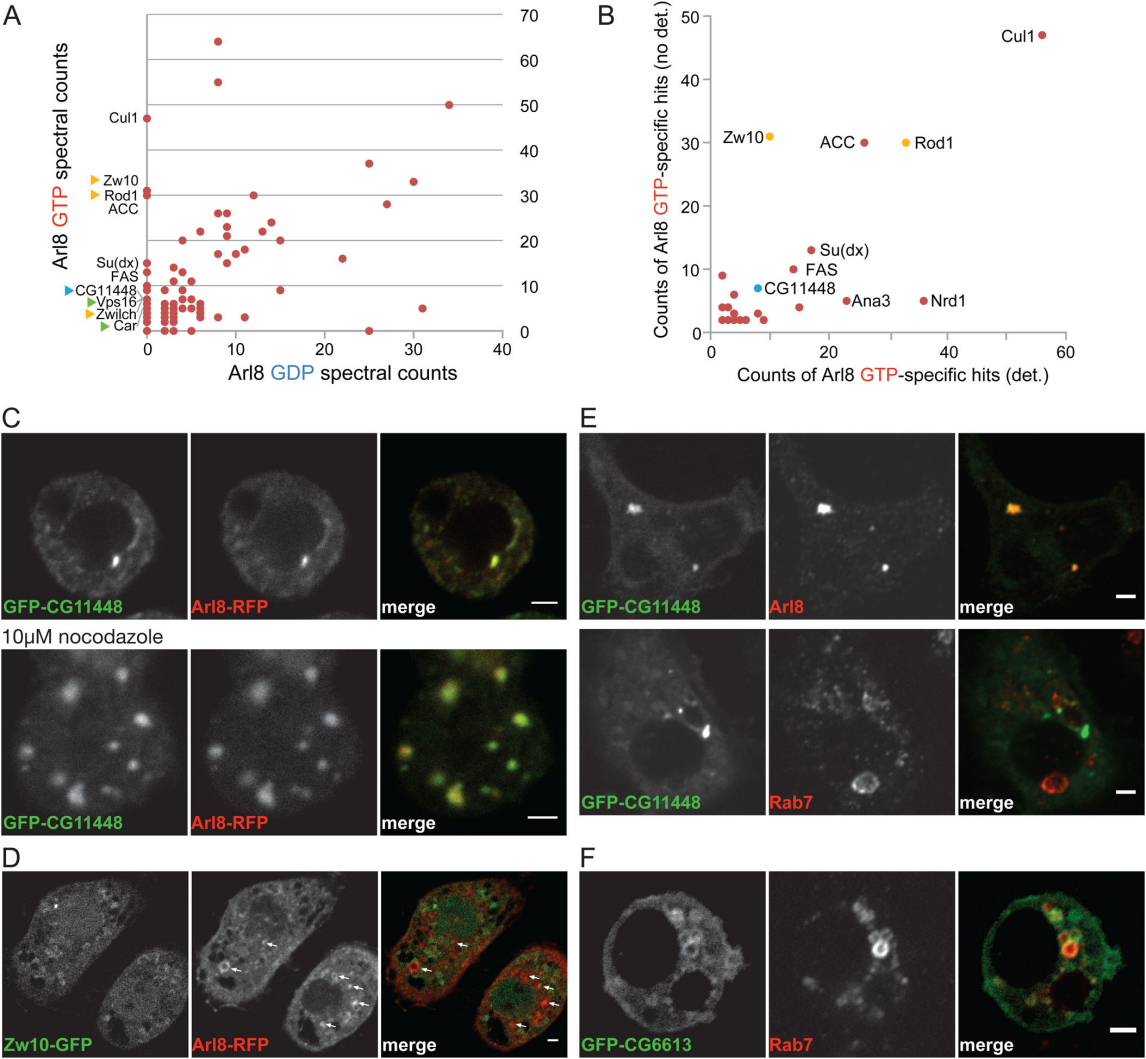
**Identification of Arl8 interacting proteins by affinity chromatography.** (A) Comparison of the spectral counts of proteins isolated by affinity chromatography using GST-Arl8 with mutations that lock the protein in either a GDP or GTP conformations. S2 cell lysates were prepared without detergent. Abundant GTP-specific interactors are labelled: yellow triangles indicating subunits of the NRZ complex, green triangles indicating two subunits of the HOPS complex [Carnation (Car) being Vps33]. Further HOPS subunits [Deep orange (Vps18), Vps11 and Vps39] were present but with fewer spectra (two with GTP, zero with GTP). CG11448 (blue triangle) is the *Drosophila* ortholog of mammalian RILP. Cullin 1 (Cul1), Acetyl-CoA carboxylase (ACC), Suppressor of deltex [Su(dx)] and Fatty acid synthase (FAS) are also shown. Not shown are hsp70, tubulin, Rme-8 and Arl8 itself, none of which were GTP-specific but whose spectral counts exceeded the axis scales. A full list of proteins and spectral counts from the purifications is in Table S1. (B) Comparison of the spectral counts for the proteins found exclusively with the GTP-locked form of Arl8 in both the purification in (A) and in a purification using S2 cell lysates prepared using the detergent CHAPS. See also Table S1. (C) Confocal micrographs of *Drosophila* S2 cells co-expressing Arl8-RFP and GFP-CG11448. In the lower row the cells were treated with 10 μM nocodazole for 2 h prior to fixation to depolymerise microtubules. (D) Confocal micrograph of S2 cells co-expressing Arl8-RFP (arrows) and Zw10-GFP. (E) Confocal micrographs of S2 cells expressing GFP-CG1148 and stained with antibodies against Arl8 or Rab7. (F) Confocal micrograph of S2 cells expressing GFP-CG6613 and stained with antibodies against Rab7. For panels B-E, at least six images were obtained for each condition with representative examples shown. Scale bars: 2 μm.

The above phenotypes indicate that Arl8 is involved in both the late endocytic pathway and also long-range transport in neurons. This suggests that, as with other GTPases and with Arl8 in other systems, *Drosophila* Arl8 recruits several different effectors to lysosomal membranes which exert different roles. To characterise the binding partners for *Drosophila* Arl8 we performed affinity chromatography with recombinant GST-Arl8 carrying Q75L or T34N mutations. These mutations lock the protein in either the GTP-bound active conformation (Q75L) or an inactive conformation (T34N) (Garg et al., 2011; Hofmann and Munro, 2006; Nakae et al., 2010). Lysates were prepared from the S2 cell line with or without the detergent CHAPS, and applied to beads coated with GST-Arl8. After washing, bound proteins were eluted and identified by mass-spectrometry. Comparing the proteins that bound to the GTP and the GDP forms of Arl8 revealed several that were specific to the active GTP-bound form (Fig. 4A; Table S1). These include Vps11, Vps16A, Deep orange (Vps18), Carnation (Vps33) and Vps39; *Drosophila* orthologs of five of the six subunits of the HOPS complex that is a known effector for Arl8b in mammalian cells (Garg et al., 2011; Khatter et al., 2015a). In contrast, we did not find spectra from CG6613, the *Drosophila* ortholog of PLEKHM1 that in mammals has been reported to interact with Arl8b, but not Arl8a (Marwaha et al., 2017). Mammalian PLEKHM1 also binds Rab7, and our previous affinity chromatography with *Drosophila* Rab7 readily detected an interaction with CG6613 (Gillingham et al., 2014; Tabata et al., 2010).

Of the other GTP-specific proteins, several have been linked to membrane traffic in mammalian cells but not previously linked to Arl8. These include Rod, Zw10 and Zwilch that are subunits of the NRZ (NAG:RINT1:ZW10) complex that resides in the ER and has been proposed to act as a tether for both Golgi-to-ER vesicles and lipid droplets (Çivril et al., 2010; Wainman et al., 2012; Xu et al., 2018). Another GTP-specific protein with a link to membrane traffic is CG11448, the single *Drosophila* ortholog of the mammalian protein RILP (Rab interacting lysosomal protein), and its paralogs RILPL1 and RILPL2. RILP is a Rab7 effector that recruits the dynein motor protein to late endosomes and lysosomes, whilst RILPL1 and RILPL2 are less well characterised but appear to act in ciliogenesis (Cantalupo et al., 2001; Jordens et al., 2001; Schaub and Stearns, 2013; Wang et al., 2004). GTP-specific binding to Arl8 was seen for the NRZ subunits and CG11448 both with and without detergent used in lysate preparation (Fig. 4B). Several other proteins appeared specific for the GTP-bound form of Arl8 but they are abundant cytosolic enzymes or nuclear proteins with no known link to membrane traffic and since they seem less likely to be physiological interactors they were not investigated further.

### The *Drosophila* ortholog of RILP interacts with Arl8

In order to investigate whether some of the putative interaction partners described above bind directly to *Drosophila* Arl8, we initially compared the localisation of GFP-tagged versions of the protein in S2 cells with that of Arl8-RFP. CG11448, the *Drosophila* ortholog of RILP, co-localised with Arl8-RFP, with both proteins showing a striking concentration into clusters in the perinuclear region (Fig. 4C). This suggests that CG11448 is recruited to Arl8-containing membranes and causes them to cluster near the nucleus. In contrast to CG11448, the NRZ complex subunit Zw10 did not detectably accumulate on Arl8-RFP positive structures suggesting that if these proteins do interact *in vivo*, then the interaction is not strong enough to control the distribution of Zw10, or only occurs under particular circumstances (Fig. 4D). We thus decided to not pursue this putative interaction partner further in this study.

To investigate the possible interaction between Arl8 and CG11448 further, we next compared GFP-CG11448 to both endogenous Arl8 and the late endosomal Rab, Rab7. Endogenous Arl8, but not endogenous Rab7, accumulated on the perinuclear clusters formed by GFP-CG11448 (Fig. 4E). Rab7 remained on the characteristic large late endosomes which are also positive for its known effector CG6613, the *Drosophila* ortholog of PLEKHM1 (Fig. 4F). Thus it appears that CG11448 interacts with Arl8-containing membranes but not those positive for Rab7.

### CG11448 localisation is dependent on Arl8 and an intact microtubule network

**Fig. 5.**
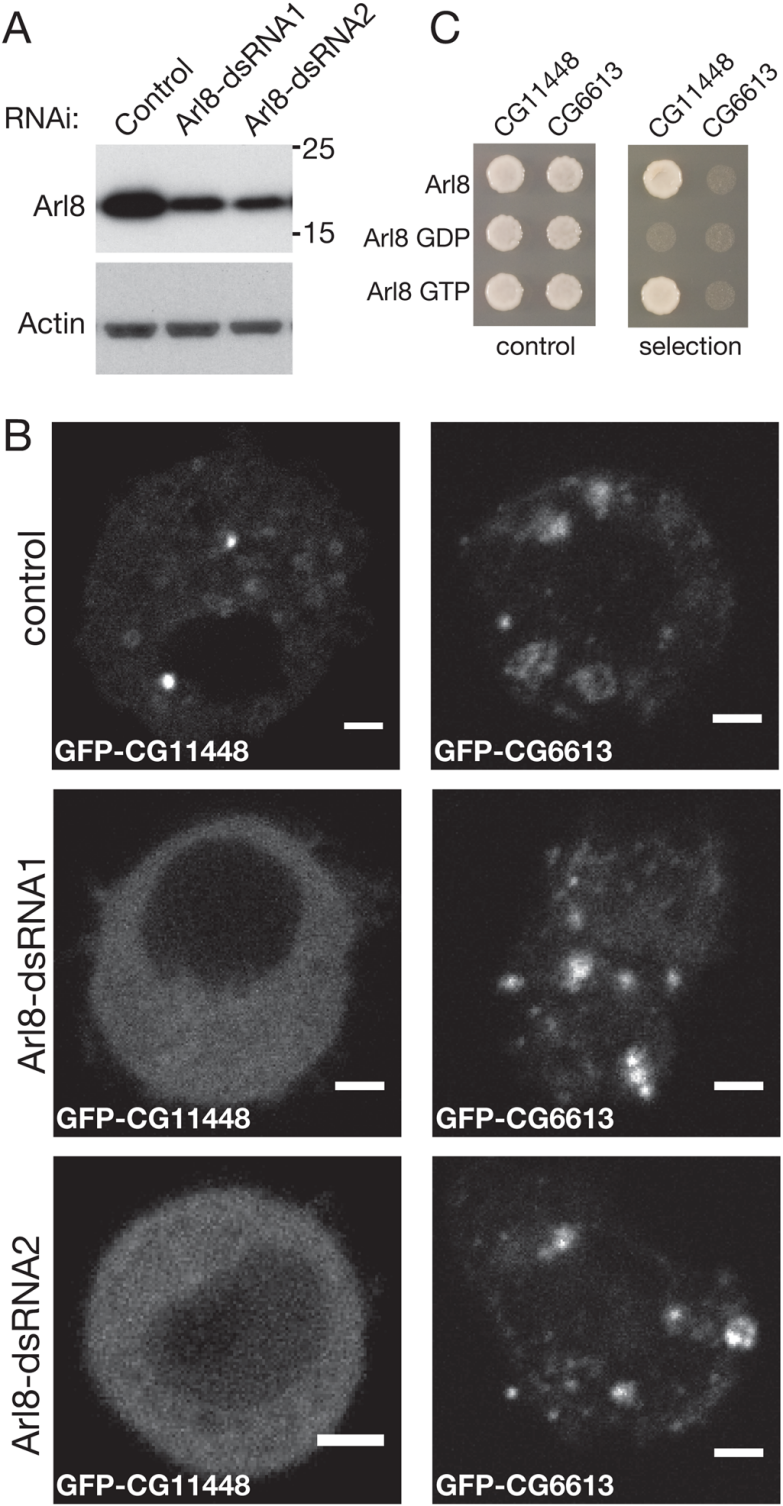
**Arl8 recruits the *Drosophila* ortholog of RILP to lysosomes.** (A) Immunoblots of lysates from cells treated with control dsRNA, Arl8-1 (dsRNA1) and Arl8-2 (dsRNA2). The blots were probed for Arl8 and for β-actin as an internal control. (B) Confocal micrographs of *Drosophila* S2 cells expressing GFP-CG1148 or GFP-CG6613 and treated with control dsRNA or two different dsRNAs against Arl8 (dsRNA1 and dsRNA2) as indicated. GFP-CG11448 and GFP-CG6613 were punctate in the majority of cells imaged (80% of 21 and 56% of 16 respectively) with higher expression levels giving a diffuse distribution. After RNAi the punctate distribution for CG11448 was reduced to 12% of 25 (dsRNA1) and 23% of 39 (dsRNA2), whereas CG6613 was not substantially affected with punctate distribution, seen in 40% of 15 (dsRNA1) and 63% of 11 (dsRNA2). Scale bars: 2 μm. (C) Yeast-two-hybrid interactions between the indicated forms of Arl8 and the proteins CG11448 and CG6613.

To characterise further the role of Arl8 in CG11448 function, we investigated whether the localisation of CG11448 was affected by knocking down endogenous Arl8 with RNAi. Two independent dsRNAs (dsRNA1 and dsRNA2) both substantially decreased the level of endogenous Arl8 (Fig. 5A). Knockdown of Arl8 resulted in GFP-CG11448 becoming diffuse, suggesting that Arl8 is the main means by which CG11448 is recruited to membranes (Fig. 5B). In contrast, reducing the levels of endogenous Arl8 with either of the dsRNAs did not cause a detectable change in the distribution of the late endosomal marker GFP-CG6613. These results suggest that Arl8 recruits GFP-CG11448 to lysosomal membranes, and consistent with this being a direct interaction we found that CG11448 interacts with the GTP-bound form of Arl8 in a yeast two-hybrid assay (Fig. 5C).

As noted above, GFP-CG11448 not only co-localises with Arl8 but also accumulates in clusters near the nucleus, suggesting that its over-expression results in relocation of Arl8-containing membranes, analogous to the relocation of lysosomes to the cell periphery seen in mammalian cells overexpressing the Arl8-binding kinesin adaptor SKIP/PLEKHM2 (Rosa-Ferreira and Munro, 2011). To test if this distribution requires not just Arl8 but also microtubule-based transport, we treated S2 cells expressing either GFP-CG11448 or Arl8-RFP or both, with nocodazole to disrupt microtubules. We found that microtubule disruption caused GFP-CG11448 to become dispersed, but it still co-localised with Arl8-RFP (Fig. 4C). This indicates that the recruitment of extra CG11448 to lysosomes by Arl8 has the effect of directing their movement towards microtubule minus-ends at the perinuclear region.

## DISCUSSION

This initial characterisation of the function of *Drosophila* Arl8 indicates that the protein has similar roles to those played by the previously characterised orthologs from mammals and *C. elegans*, and thus *Drosophila* should be a useful tractable model to study Arl8 biology. In particular the *Drosophila* protein has a role in the function of the late endosomal pathway which is likely to reflect, at least in part, an interaction with the HOPS complex that acts as a tether in various fusion steps in this pathway. Our affinity chromatography with Arl8 also reproducibly found an interaction with a second tethering complex, the NRZ complex of the ER. Our preliminary analysis did not provide clear evidence for a strong interaction *in vivo*, but it is not inconceivable that Arl8 could act to tether lysosomes to the ER as has been seen for the small GTPase, Rab18, that is able to tether lipid droplets to the ER via an interaction with the NRZ complex (Gillingham et al., 2014; Xu et al., 2018).

Our studies also provide evidence for a role for *Drosophila* Arl8 in long-range transport in axons, similar to the findings from studying mutants in the *C. elegans* ortholog of *Arl8* (Klassen et al., 2010; Niwa et al., 2016). In mammals and *C. elegans* Arl8 has also been shown to be involved in controlling the intracellular distribution of lysosomes by linking them to microtubule-dependent motor proteins (Guardia et al., 2016; Klassen et al., 2010; Rosa-Ferreira and Munro, 2011). Consistent with this, we found evidence for *Drosophila* Arl8 binding to CG11448, the single *Drosophila* ortholog of the mammalian dynein adaptor RILP and its two paralogs RILPL1 and RILPL2 that are less well characterised. In mammals, RILP binds to Rab7 and also to the p150^Glued^ subunit of the dynactin complex that activates dynein (Cantalupo et al., 2001; Jordens et al., 2001). RILP has also been reported to interact with the HOPS complex, but our findings suggest that the interaction we see between Arl8 and CG11448 is direct, although of course this would not preclude CG11448 also binding to HOPS. Residues in mammalian RILP that bind to Rab7 have been identified by structural studies, and these comprise a unique region that is missing from both human RILPL1/2 and *Drosophila* CG11448 (Wang et al., 2004; Wu et al., 2005). This suggests that the ability of RILP to bind Rab7 is a vertebrate-specific feature that was acquired by RILP after the gene family expanded in vertebrates. In contrast the p150^Glued^-binding region in RILP is well conserved in both RILPL1 and CG11448 (Johansson et al., 2007). We did not detect dynein or dynactin amongst the proteins binding to Arl8 by affinity chromatography, but their binding to adaptors is known to be reversible and previous isolation of a known dynein adaptor found a similar lack of detectable dynein following affinity chromatography (Dienstbier et al., 2009). We tested the ability of human Arl8b to bind to all three members of the human RILP family by co-overexpression but saw no detectable enrichment of the RILP family members on Arl8b-positive lysosomes (C.R.-F., S.T.S. and S.M., unpublished).

Further work will be needed to determine if the requirement for Arl8 for normal neuronal transport reflects an interaction with CG11448, and indeed proving a requirement for a particular GTPase:effector interaction is not trivial. The phenotype we observed of synaptic membrane proteins accumulating in axons is similar to that observed in *Drosophila* mutants with perturbations in dynein function (Martin et al., 1999). Although it is possible that this accumulation reflects a defect in retrograde transport as microtubules are polarised in axons with their minus-ends at the cell body, there is good evidence from many systems that plus-end and minus-end transport are tightly coupled (Encalada et al., 2011; Haghnia et al., 2007). Thus defects in dynein-based transport could indirectly affect anterograde transport. In other systems, Arl8 has also been observed to directly stimulate kinesin-based transport, by either binding kinesin directly or in mammals through the linker protein PLEKHM2 (Guardia et al., 2016; Rosa-Ferreira and Munro, 2011; Wu et al., 2013). *Drosophila* lack a clear ortholog or PLEKHM2, with the most closely related PH-domain containing protein, CG17360, lacking the RUN domain that binds Arl8 and kinesin light chain in PLEKHM2, and previous work has indicated that CG17630 instead binds Rab39 (Gillingham et al., 2014). *Drosophila* Arl8 could still recruit one or more kinesins by direct binding or via an as yet identified linker, but it is at least possible that the defects we observe in axons in the absence of Arl8 reflect defects in dynein-based transport mediated by RILP.

Clearly further work will be required to determine if there are further Arl8 effectors in *Drosophila*, and which of these effectors is responsible for which aspects of its function. However our studies do indicate that *Drosophila* should prove a tractable and relevant model system with which to gain new information about Arl8 that is also relevant to mammals.

## MATERIALS AND METHODS

### *Drosophila* stocks, mutagenesis and genetics

Fly stocks were kept at 25°C unless otherwise stated. A stock carrying PBac{RB}Gie^e00336^ was obtained from the Bloomington *Drosophila* Stock Center (ref. 17846). A FRT82B PBac{RB}Gie^e00336^ (*Arl8^KO1^*) stock was created by meiotic recombination of the two alleles. A stock was recovered that was resistant to geneticin (G418, confirming the presence of the FRT element) and was lethal over *Arl8^KO1^*. The absence of detectable Arl8 in homozygous larvae was then confirmed by immunoblotting. Mitotic recombination between FRT sites was achieved by expression of flippase (hs FLP22), induced by heat shock at 37°C for 1-2 h at different stages of *Drosophila* development. The transgenes used for this purpose, in addition to P{neoFRT}82B PBac{RB}Gie^e00336^ (FRT82B *Arl8^KO1^*), were P{neoFRT}82B, P{GFPnls}; P{neoFRT}82B, Ubi P{mRFPnls}, with P{neoFRT}82B P{w[+mC]=ovoD1-18}3R used for generation of germline clones.

pUAST Arl8 and pUAST Arl8-GFP transgenic lines were obtained by germline transformation (BestGene Inc., Chino Hills, USA) and mapping with a double balancer line. The other transgenes used were P{W^+^ UAS-GFP Lamp1}/Cyo (Pulipparacharuvil et al., 2005), and P{w+, UASp-YFP.dRab7}/SM5 (Bloomington *Drosophila* Stock Center). The Arl8 RNAi line used was obtained from Vienna *Drosophila* Resource Center (line 26085). Immediately before imaging, wandering third instar larvae were selected based on their genotypes, quickly rinsed in ddH_2_O to remove any debris and then moved to egg-laying petri dishes.

### Cell culture, transfection and RNAi

*Drosophila* S2 cells (D.MEL-2, Life Technologies) were validated as being of *Drosophila* origin using species-specific antibodies. They were maintained in Express Five SFM medium supplemented with 1% L-glutamine (Thermo Fisher Scientific) and penicillin/streptomycin at 25°C. S2 cells were transfected with 1 µg of DNA plasmid, including a carrier plasmid (pAW empty vector), in 6-well plates using 5 μl of polyethylenimine (1 μg/μl).

dsRNA against Arl8 or luciferase (control) were transcribed *in vitro* with T7 RNA polymerase (T7 RiboMAX Express RNAi System, Promega). The Arl8 dsRNA1 corresponded to residues 1-197 of the open reading frame (ATGTTG…TGTGAC), and dsRNA2 to residues 250-450 (CGCTATT…TCTATCG). S2 cells seeded in 6-well plates were transfected with 30 µg of dsRNA and 20 μl of TransFast (1 μg/μl, Promega), and cell analysed four days later.

### Affinity chromatography

GST-Arl8Δ17 (lacking the first 17 residues that comprise to the N-terminal amphipathic helix) with the mutations T34N and Q75L, that lock the protein in a GDP- or GTP-bound conformation were produced in *Escherichia coli* BL21-GOLD (DE3). Bacteria were dounce homogenised and sonicated in 20 mM Tris-HCl, pH 8, 110 mM KCl, 5 mM MgCl_2_, 5 mM β-mercaptoethanol, protease inhibitors, with or without 1% CHAPS (lysis buffer with or without CHAPS) at 4°C, and bound to Glutathione-Sepharose beads (GE Healthcare). For large-scale affinity chromatography, S2 cells were dounce homogenised in lysis buffer, with or without CHAPS at 4°C, passed through a 30 gauge needle and the clarified cytosol was incubated with GST-Arl8 coated beads in the presence of GDP or GTPγS (Sigma-Aldrich). Samples were eluted with a high concentration of salt with the addition of the opposite nucleotide.

### Yeast two-hybrid analysis

Bait plasmids were cloned into pDEST32 (Invitrogen) and transfected into the yeast strain PJ69-4a (James et al., 1996). Prey fragments were cloned into pDEST22 (Invitrogen) and transfected into the yeast strain PJ69-4α. Strains were mated, grown for 24 h in YEPD, and replicates grown on selective medium at 30°C for 3 days.

### Antibodies and immunoblotting

The antibodies used here were rabbit anti-β actin (ab8227; Abcam), mouse anti-GFP (11 814 460 001; Roche), rabbit anti-Rab7 (Tanaka and Nakamura, 2008), rabbit anti-Arl8 (Hofmann and Munro, 2006), Cy3-conjugated anti-HRP (123-165-021; Jackson ImmunoResearch, Ely, UK), rabbit anti-Syt (West et al., 2015), mouse anti-BRP [nc82; Developmental Studies Hybridoma Bank (DSHB)], mouse anti-Notch extracellular domain (C458.2H; DSHB). Information on validation of the antibodies is available from the commercial source or the indicated publication. Primary antibodies were detected by secondary antibodies conjugated with Alexa fluorochromes (Thermo Fisher Scientific) or with HRP (DAKO).

Whole flies, larvae or the indicated dissected fly tissues were lysed in equivalent amounts of SDS sample buffer and insoluble debris was removed by brief centrifugation. S2 cells growing in plates were dissolved directly in SDS sample buffer. Protein extracts were separated in 4–20% Tris-Glycine gels (Invitrogen), transferred to PVDF membranes and probed with primary and HRP-conjugated secondary antibodies, detected by chemiluminescence (ECL; GE Healthcare).

### Fluorescent imaging

S2 cells were fixed in 4% formaldehyde in PBS for 15 min, permeabilised and blocked with PBS, 0.1% Triton X-100 and 10% FCS for 1 h. L3 larval salivary glands, imaginal discs and the fat body were dissected in PBS and fixed in 4% formaldehyde for 30 min, before permeabilisation and blocking in PBS, 0.3% Triton X-100, 20% FCS. Egg chambers were fixed in 8% formaldehyde and permeabilised and blocked in PBS, 0.1% Tween-20, 20% FCS. Dissection of larval motor neurons and neuromuscular junctions was performed in PBS on a Sylgard plate, fixed in 4% formaldehyde for 7 min, permeabilised in PBS, 0.1% Triton X-100 and 10% FCS. Tissue culture cells or fly tissues were probed with primary antibodies, or conjugated anti-HRP, in the same solution used for blocking and permeabilisation and then labelled with Alexa Fluor-conjugated secondary antibodies (Invitrogen) and mounted in Vectashield (Vector Laboratories, Peterborough, UK).

For LysoTracker or Magic Red staining, L3 imaginal discs or the fat body were quickly dissected out in Shields and Sang M3 medium (Sigma-Aldrich), 2% FBS, stained with either 100 nM of LysoTracker Red DND-99 (1 mM stock; Thermo Fisher Scientific) or with a 1:10 dilution of Magic Red stock solution (Bio-Rad) in Shields and Sang M3 medium, 2% FBS for 5-10 min. After washing in Shields and Sang M3 and 2% FBS, the live tissues were transferred to 2.5% methyl cellulose in Shields and Sang M3 medium in 35 mm glass-bottomed tissue culture dishes (FluoroDish; World Precision Instruments, Hitchin, UK). Images were obtained with LSM710 or LSM780 confocal microscopes (Zeiss, Cambridge, UK), and Fiji (ImageJ) was used for image analysis and quantitation procedures (Schindelin et al., 2012).

## Supplementary Material

Supplementary information

First Person interview
