# Supplementary material for: The small G protein Arl8 contributes to lysosomal function and long-range axonal transport in Drosophila
Source: Biol Open. 2018 Aug 20;7(9):bio035964. doi: 10.1242/bio.035964 (PMC6176938; doi:10.1242/bio.035964)
Supplement: Supplementary information [file biolopen-7-035964-s1.pdf]

© 2018. Published by The Company of Biologists Ltd | Biology Open (2018): doi:10.1242/bio.035964: Supplementary information

FIRST PERSON

First person – Cláudia Rosa-Ferreira

First Person is a series of interviews with the first authors of a selection of papers published in Biology Open, helping early-career researchers promote themselves alongside their papers. Cláudia Rosa-Ferreira is first author on 'The small G protein Arl8 contributes to lysosomal function and long-range axonal transport in *Drosophila*', published in BiO. Cláudia is a Business Development Associate at CMR Surgical, and previously worked in the lab of Sean Munro at MRC Laboratory of Molecular Biology, Cambridge, UK, investigating how lysosomes govern important cellular activities and respond to external cues such as plasma membrane repair, tumour invasion and metabolic signalling.

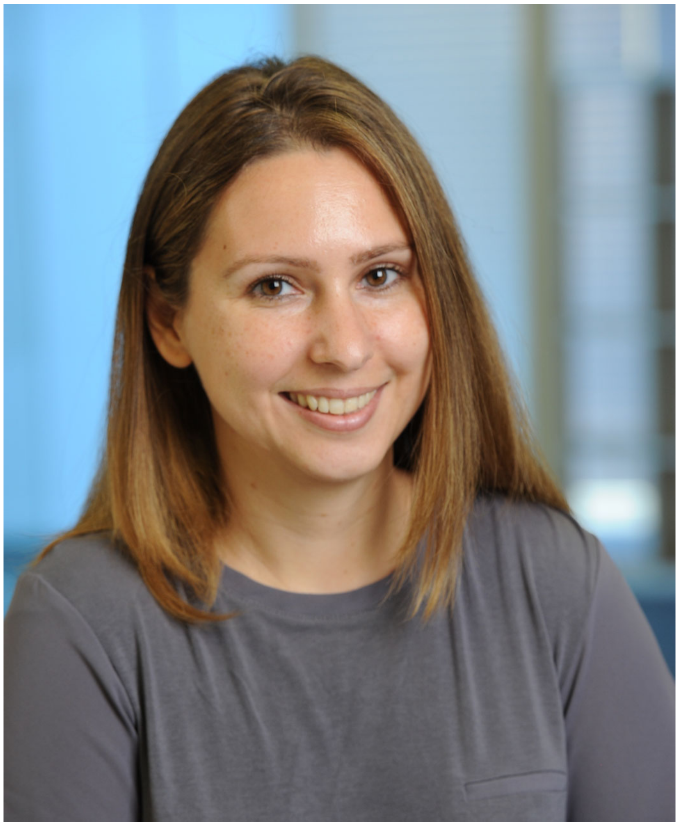

Cláudia Rosa-Ferreira

What is your scientific background and the general focus of your lab?

Before joining the Sean Munro lab to work on membrane trafficking, I worked in cancer research both in my home country, Portugal, and then in Cambridge, UK. I worked on trying to understand whether cells with a certain identity were more prone to undergoing tumorigenesis. I also worked on understanding the process by which centrosomes duplicate and on the different cell cycle roles of isoforms of a kinase are required for asymmetric cell division in *Drosophila*.

Sean's lab focuses on understanding the mechanism by which transient interactions between G proteins and their effectors enable efficient cargo traffic which support functions such as cellular signalling, cell migration and actin cytoskeleton remodelling. The Golgi complex is the hub for the sorting of cargo that needs to be secreted or delivered to the endocytic pathway, while contributing to the recycling and redistribution of key factors that operate at different cellular compartments to maintain cell homeostasis. Recent reports from Sean's lab have shed light on these mechanisms by showing that the Golgin proteins contribute to this sophisticated sorting mechanism and by identifying novel effectors for Rab and G proteins.

How would you explain the main findings of your paper to non-scientific family and friends?

Arl8 is a fascinating protein that seems to be able to fine tune the positioning and function of a cellular organelle called the lysosome with extreme precision and sophistication. Lysosomes are known to break down unwanted cellular factors and recycle them into their basic components, by dynamically fusing with another type of cellular organelle called late endosomes, which have previously sorted these unwanted factors. Arl8 is found at the surface of lysosomes, where it governs lysosomal function through interaction with various proteins.

We found that removing Arl8 from fruit flies means that they cannot live, and this is reverted by adding Arl8 back to motor neurons, although the flies were unable to fly. On the other hand, fruit fly larvae without Arl8 move slowly as they present paralysis of their most distal segments. We then looked at motor neurons without Arl8, and found they were defective. The transport of neuronal factors that are required at the synapse was affected in the motor neurons lacking Arl8 and their synapses were smaller. This indicated that the larval paralysis is due to deficient stimulation of muscle fibres by the defective motor neuron synapses.

In addition, we found that in fruit fly cells lacking Arl8, late endosomes and lysosomes tend to accumulate and that the breakdown of unwanted cellular factors is compromised. As Arl8 functions are likely to reflect its interaction with other cellular factors, we set out to investigate its ligands. We found a novel interaction with a protein linked to cellular transport in humans. Additional work is now required to understand whether Arl8's interaction with this factor is responsible for any of the defects that we found in fruit flies.

"Arl8 is a fascinating protein that seems to be able to fine tune the positioning and function of [...] the lysosome with extreme precision and sophistication."

What are the potential implications of these results for your field of research?

In this work, we found that in *Drosophila*, Arl8 is required for delivery of cargo to be degraded in lysosomes, in line with reports in mammalian cells and *C. elegans* that implicate Arl8 in the fusion

Cláudia Rosa-Ferreira's contact details: CMR Surgical, Unit 2, Crome Lea Business Park, Madingley Road, Cambridge, CB23 7PH, UK.
E-mail: csdrferreira@gmail.com

between late endosomes and lysosomes, possibly through an interaction with the HOPS complex. Motor neurons lacking Arl8 accumulate markers of synaptic vesicles at the axon and display smaller synapses, which are likely to cause larval distal paralysis; this is consistent with observations in *C. elegans*, where Arl8 mutation caused disruption in axonal transport.

Lysosomal positioning is a tightly regulated process that is the result of responses to various stimuli and is key to lysosomes performing their multiple functions. In previous work, we found that in human cells, anterograde movement of lysosomes is dependent on Arl8 interaction with the effector protein SKIP which binds to kinesin-1. In this paper, however, we found that in *Drosophila*, where SKIP is not conserved, Arl8 binds in a GTP-dependent manner to CG11448, an RILP-like protein homologue which causes Arl8 to relocate to a juxtanuclear position, dependent on an intact microtubule cytoskeleton.

The novel interaction between Arl8 and CG11448 implies that Arl8 directs retrograde movement of lysosomes in *Drosophila*. Nevertheless, it is not known whether this interaction is responsible for Arl8 functions in the regulation of endocytic traffic to lysosomes and of axonal transport in motor neurons. This finding indicates that Arl8 displays a conserved role in governing the intracellular distribution of lysosomes via interaction with effector proteins that are adaptors of microtubules motor proteins. However, more work is necessary to understand whether Arl8 regulates the retrograde movement of lysosomes in humans, or if the mechanisms by which Arl8 regulates lysosomal movement and function have evolved to target plus-end-motors in mammals.

What's next for you?

Having spent 10 years doing basic research, where I had the opportunity to address many fundamental questions, I wanted to learn more about the process of developing and commercialising diagnostic and therapeutic solutions, ultimately with the aim of creating real-world impact and improving healthcare. I currently work at CMR Surgical which is commercialising the next-generation surgical robot to assist in key-hole surgery. It is very rewarding to be working with a great team on a medical solution that will reach millions of patients worldwide and that will improve patient access to minimal-access surgery, thereby improving patient outcomes.

Reference

Rosa-Ferreira, C., Sweeney, S. T. and Munro, S. (2018). The small G protein Arl8 contributes to lysosomal function and long-range axonal transport in *Drosophila*. *Biol. Open* **7**: bio035964.
